# A Quantitative Synthesis of Eight Decades of Global Multiple Sclerosis Research Using Bibliometrics

**DOI:** 10.3389/fneur.2022.845539

**Published:** 2022-02-24

**Authors:** Ismail Ibrahim Ismail, Mohammed Saqr

**Affiliations:** ^1^Department of Neurology, Ibn Sina Hospital, Kuwait City, Kuwait; ^2^School of Computing, University of Eastern Finland, Joensuu, Finland

**Keywords:** multiple sclerosis, bibliometrics, articles, citations, impact factor, scientific collaboration, publication trends, country productivity

## Abstract

Bibliometric studies on the field of multiple sclerosis (MS) research are scarce. The aim of this study is to offer an overarching view of the body of knowledge about MS research over eight decades–from 1945 to 2021–by means of a bibliometric analysis. We performed a quantitative analysis of a massive dataset based on Web of Science. The analysis included frequencies, temporal trends, collaboration networks, clusters of research themes, and an in-depth qualitative analysis. A total of 48,356 articles, with 1,766,086 citations were retrieved. Global MS research showed a steady increase with an annual growth rate of 6.4%, with more than half of the scientific production published in the last decade. Published articles came from 98 different countries by 123,569 authors in 3,267 journals, with the United States ranking first in a number of publications (12,770) and citations (610,334). A co-occurrence network analysis formed four main themes of research, covering the pathophysiological mechanisms, neuropsychological symptoms, diagnostic modalities, and treatment of MS. A noticeable increase in research on cognition, depression, and fatigue was observed, highlighting the increased attention to the quality of life of patients with MS. This bibliometric analysis provided a comprehensive overview of the status of global MS research over the past eight decades. These results could provide a better understanding of this field and help identify new directions for future research.

## Introduction

Multiple sclerosis (MS) is a chronic autoimmune disorder of the central nervous system (CNS) characterized by inflammation, demyelination, followed by neurodegeneration. MS typically presents in young adults in the third or fourth decade of life, with females two times as likely to be affected (1). MS is one of the leading causes of neurological disability in young adults, resulting in remarkable socioeconomic impacts and the need for lifetime support and management (2). A total of 2.8 million people are estimated to have MS worldwide (3). The prevalence of MS in the population has shown a 30% increase in 2020, compared to 2013, in every world region. The 2020 global prevalence of MS is 35.9 per 100,000 people, while the pooled incidence rate is 2.1 per 100,000 persons/year (3).

Since the first modern clinical description of a case of MS in the medical literature by Ollivierd' Angers in 1824 (4), there has been a considerable growth in scientific publications in the field of MS research (5). In the last few decades, technological advances in genetics, molecular medicine, pathology, and imaging had encouraged researchers all over the world to publish a vast amount of papers, aiming at a better understanding of the etiology, pathogenesis, clinical features, diagnosis, and treatment of MS (6).

The number of articles and their citations are considered a strong indicator of the importance of this disease. However, there has been little examination of the precise publication patterns and characteristics in MS, and bibliometric studies are rare. Aleixandre-Benavent and his colleagues (7) presented the only global bibliometric analysis of MS research from 2003 to 2012 to the best of our knowledge, while Espiritu and colleagues (8) examined the scientific impact of MS and Neuromyelitis Optica spectrum disorder research in Southeast Asia. The most recent analysis has been an Altimetric study based on the top 100 discussed papers over social media (9).

Bibliometrics is an interdisciplinary science that uses mathematical and statistical methods to analyze and quantitatively evaluate the contribution and productivity of a research field, including different countries, institutions, journals, or authors. Bibliometric studies have been used to provide a clear presentation of publication characteristics, hotspots, and research trends in a specific field to help guide policy decision-making (10). In doing so, bibliometrics allow an overarching view of a field of knowledge, the creators (authors and countries), the dissemination outlets (e.g., journals), the themes of knowledge, and their evolution (11). There is, of course, a tradeoff between inclusivity of thousands of articles (e.g., in bibliometrics) and in-depth fine-grained evaluation (e.g., in systematic reviews). Both types of scientific synthesis serve a function that is rather complementary.

In this work, we conducted a bibliometric analysis of the published literature related to MS research included in the Web of Science databases (WoS), over the past eight decades worldwide, which has not been performed so far, to our knowledge. This bibliometric analysis provides insight into the core body of knowledge about MS, the creators, the way it was disseminated, and the trends in MS research to have a better understanding of the current research, impact, gaps, and possible future scientific research.

## Methods

The data were retrieved from the Web of Science (WoS) database on April 29, 2021 using the query “multiple sclerosis” OR “disseminated sclerosis.” WoS offers a robust database with curated sources that are subject to rigorous quality control and therefore, ensures that all journals are of good scientific integrity (12). Only original English articles that were peer-reviewed were retrieved with them with all their meta-data available in WoS. Since the data were retrieved from a single database, there were no duplicates. The data were retrieved and cleaned, so misspelled author names, names with several spellings, and special characters were fixed and combined. Keywords were cleaned with Google Openrefine, which has several natural language processing and clustering algorithms for the detection of keywords with similar spellings, e.g., (“*autoimmune disease*,” “*autoimmune diseases*,” “*auto-immune disease*,” and “*auto-immune diseases*”), all of such keywords were detected and combined. Manual combination was also performed to combine identical keywords that were not similar in spelling, e.g., (“MS,” “*multiple sclerosis*”) but had an identical meaning. This step was necessary to avoid the fragmentation of keywords, i.e., the same keyword written in different forms and therefore, could cloud the accuracy of the different counts and trends. The original keywords were retained and tabulated in the results section. Two other types of keywords were mined from the titles and abstracts to extract the most frequently recurring keywords in articles abstracts or titles.

The cleaned dataset was analyzed using *Bibliometrix* package (13). *Bibliometrix* offers a comprehensive platform for the analysis of bibliometrics data, which includes the extraction, tabulation, and calculation of the frequencies of articles, authors, keywords, countries, and citations (according to WoS). Since a manuscript has no formal country, the country was extracted according to the corresponding author of each country. For the country collaboration network, authors who are collaborating on the same article are considered connected. The network was partitioned (divided into connected clusters) using the Louvain modularity for community detection (14) and plotted using the Fruchterman Reingold layout algorithm (15). A similar network was constructed for the keywords by considering the keywords that co-occurred in the same manuscript as connected; the network was partitioned and plotted in the same way as the country network. For clarity, the topmost connected nodes were displayed using a threshold of degree centrality of 850, which left 57 nodes in the network.

The dataset statistics and frequencies were plotted using the functions provided by the *Bibliometrix* package. For keyword trends, two trends were created: (1) a simple frequency plot with the number of articles on the Y-axis and the year on the X-axis (2) a proportional plot where the fraction of the total articles published at each given year was plotted against the Y-axis.

## Results

### General Analysis

The dataset for this study spanned over eight decades during the period of 1945–2021 (Table 1). The dataset included 48,356 original research articles; the majority of which were journal articles, 46,611 (96.4%), who received a total of 1,766,086 citations. This long journey started with only seven articles in 1945 and expanded exponentially to reach 3,101 articles in 2020. Just around half of the articles (*n* = 24,424; 50.5%) were published during the last decade, which indicates that our dataset is more representative of recent research. Each of the included manuscripts was cited on average 36.5 with a yearly citation rate of 2.8, an indication of the relatively high potential for MS articles to be cited. Only 5.3% of the documents in our dataset were not cited, given that our dataset contained articles from 2020 and 2021 that were too soon to be cited; this is a very low percentage. A total of 123,569 authors contributed to the articles in our dataset, with only a small number of articles authored by a single author, 1,747 (3.7%), indicating that collaboration on articles was the rule. In the same vein, the average number of authors in the manuscript was 2.6. The average yearly growth rate of articles was 6.4 over the entire duration, 5.1 over the last decade, and 6.5 over the last two decades, indicating a steady and relatively stable rate of increase of MS research.

**Table 1:** Main information about the bibliometric dataset.

**Data set statistics**	
Timespan	1945–2021
Sources (Journals, Books, etc.)	3,228
Documents	48,356
Last decade publications	24,424
Total citations	1,766,086
Number of uncited documents	2,567
Average years from publication	13.2
Average citations per documents	36.5
Average citations per year per doc	2.836
References	613,328
Author's keywords (DE)	40,622
**Document types**	
Article	46,611
Proceedings paper	1,745
**Authorship data**	
Number of authors	123,569
Author appearances	300,639
Authors of single-authored documents	1,747
Authors of multi-authored documents	121,822
Single-authored documents	2,665
Mean authors per document	2.56
**Annual scientific production**	
Annual percentage growth rate	6.4%
Last 10 years growth rate	5.1%
Last 20 years growth rate	6.5%

### Country Analysis

**Figure 1:**
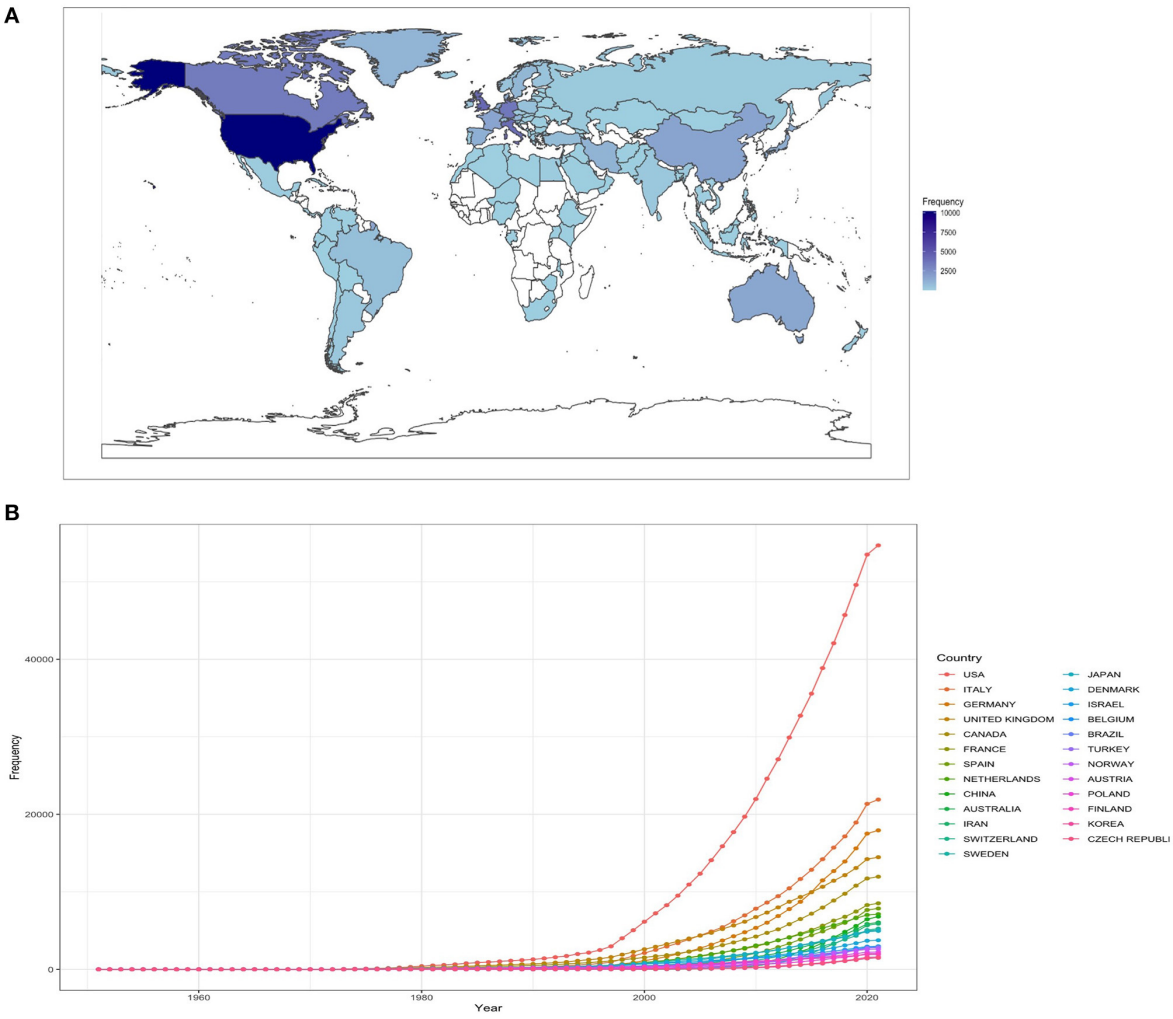
**(A)** A world map with the distribution of country productivity, **(B)** a cumulative evolution of the number of MS papers by the top 25 countries.

The published articles on MS during this period came from 98 different countries. A world map of country productivity and a list of the top 25 countries are illustrated in Figure 1. The country with the greatest number of published articles was the USA (*n* = 12,770), followed by Italy (*n* = 4,310), the United Kingdom (*n* = 3,503), Germany (*n* = 3,369), and Canada (2,404). Other countries that exceeded 1,000 manuscripts were France (*n* = 1,502), the Netherlands (*n* = 1,463), Spain (*n* = 1,437), China (*n* = 1,410), Japan (*n* = 1,203), Sweden (*n* = 1,154), Iran (*n* = 1,140), and Australia (*n* = 1,115). The country with the highest number of citations was also the USA (*n* = 610,334). Other countries that exceeded 100,000 citations were the United Kingdom (*n* = 184,932), Italy (*n* = 128,079), Germany (*n* = 116,697), and Canada (*n* = 103,437).

**Figure 2:**
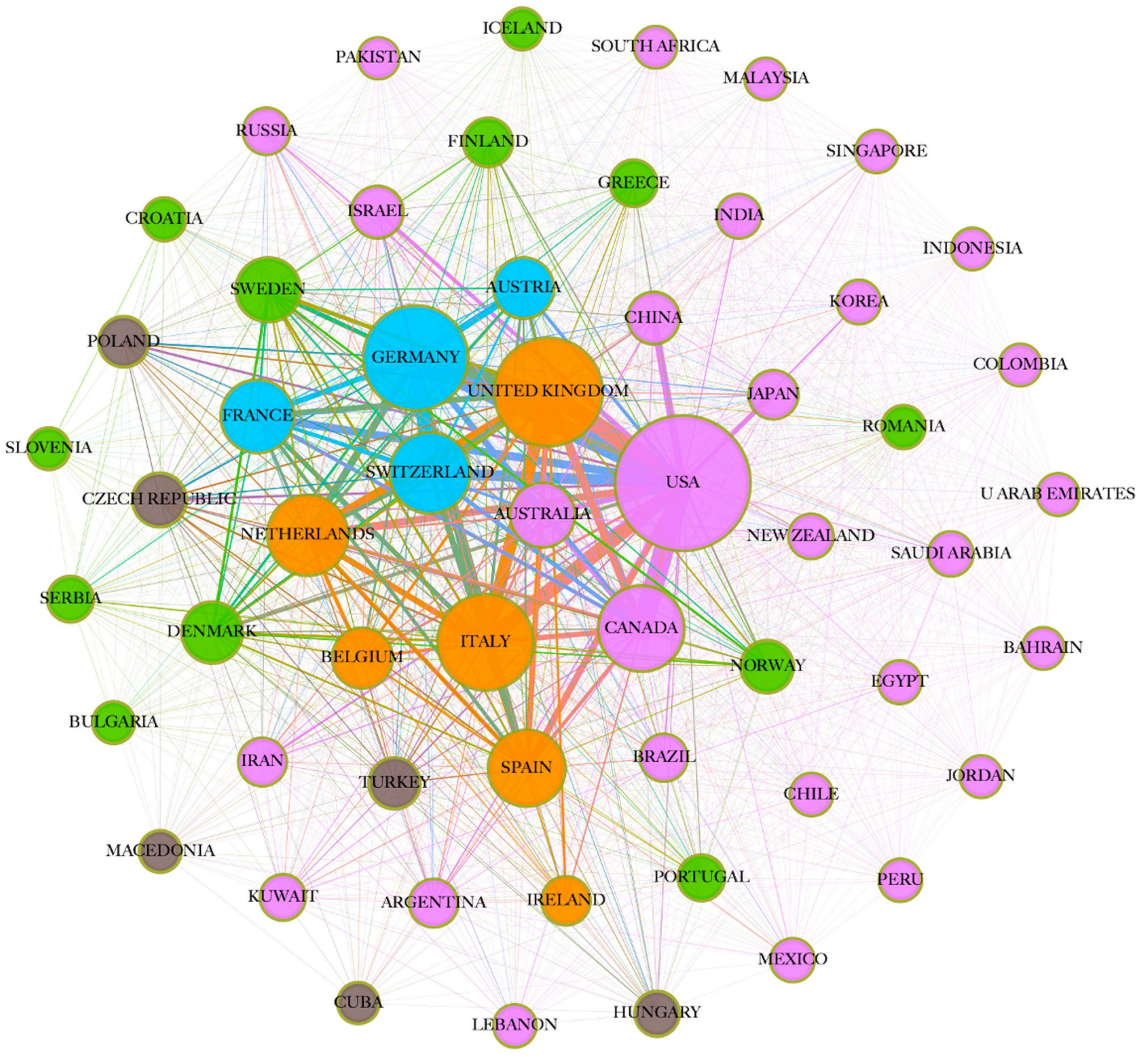
Network of international collaborations in MS-related articles (countries that frequently collaborate have a similar color; circle size indicates papers per country, and line thickness indicates the number of co-authored articles).

With regards to scientific collaboration, the USA was the leading country with a total of 10,272 single country publications (SCP), and 2,498 multiple country publications (MCP), with an MCP ratio of 0.195, which shows that the majority of the publications from the USA were single country publications. As shown in Figure 2, the network of collaboration shows a pink cluster representing developed countries who share languages, e.g., the USA, Canada, Australia, and New Zealand and their collaborators from Asia, e.g., China, Korea, Japan, India, and Singapore, and the Middle East and North Africa (MENA) region, e.g., Egypt, Kuwait, Iran, and Saudi Arabia. Another green cluster of countries is made of northern European countries, Sweden, Denmark, Finland, and their collaborators. A blue cluster is seen showing countries who have German as their official language, in addition to France. We also see an orange cluster made of other European countries, e.g., the UK, Italy, and the Netherlands. Over the last decade, several MS societies around the globe have been collaborating in data collection to accelerate research insights into innovative care and treatment for people with MS through better use of real-world data (16, 17).

Our findings could also be explained—at least partially—by epidemiological data of MS as well as country development, i.e., well-developed countries that suffered from considerable MS prevalence have shown a strong appearance in our data. The Atlas of MS (www.atlasofms.org), which is an open-source global compendium of data on the epidemiology of MS compiled by the Multiple Sclerosis International Federation (MSIF), collected epidemiologic data from 115 countries representing 87% of the world's population in 2020 (3). It has shown significant increase in MS cases, both as a proportion of their populations (MS prevalence) and in terms of growing cases (MS incidence) across North America and several European countries (the United Kingdom, Germany, Denmark). Although Europe had the highest reported incidence at 6.8, followed by the Americas at 4.8, Canada showed the highest prevalence as a country, at 168 people per 100,000, and the highest incidence, at 5.63 per 100,000 (18), while the US almost doubled its prevalence (913,925 cases) from 2013 to 2019 (19). Moreover, increasing prevalence was also reported across the MENA region, in addition to the Russian Federation, and Australia. South East Asia and Africa had the lowest reported incidence rates of 0.4 (3), which can explain the underrepresentation of these countries in our data.

### Most Cited Papers

The analysis of the most seminal articles gives an idea about the directions of the research on MS and how the community of MS researchers responded to or were influenced by certain strands of research. A total of 48 articles in literature received 1,000 citations or more. Table 2 presents the top 20 most cited articles, where all of them exceeded 1,500 citations. Among them, six articles evaluated the pathophysiological underpinnings of MS, five articles investigated MS treatments, five articles described MS diagnostic criteria or its revisions, two articles proposed severity scales, one article evaluated antibody markers to differentiate neuromyelitis optica from MS, and a single article on the role of vitamin D in immune disorders.

**Table 2:** The list of the top 20 most cited papers within our dataset.

**References**	**Publication year**	**Journal**	**Title**	**DOI**	**Citations**
Kurtzke et al. (20)	1983	Neurology	Rating neurologic impairment in multiple sclerosis. An expanded disability status scale (EDSS)	10.1212/WNL.33.11.1444	10,052
Poser et al. (22)	1983	Ann Neurol	New diagnostic criteria for multiple sclerosis: Guidelines for research protocols	10.1002/ana.410130302	6,770
Polman et al. (23)	2011	Ann Neurol	Diagnostic criteria for multiple sclerosis: 2010 revisions to the McDonald criteria	10.1002/ana.22366	5,428
McDonald et al. (24)	2001	Ann Neurol	Recommended diagnostic criteria for multiple sclerosis: Guidelines from the international panel on the diagnosis of multiple sclerosis	10.1002/ana.1032	4,985
Polman et al. (25)	2005	Ann Neurol	Diagnostic criteria for multiple sclerosis: 2005 revisions to the “McDonald Criteria”	10.1002/ana.20703	3,732
Krupp et al. (38)	1989	Arch Neurol	The fatigue severity scale. Application to patients with multiple sclerosis and systemic lupus erythematosus	10.1001/archneur.1989.00520460115022	3,218
Trapp et al. (27)	1998	N Engl J Med	Axonal Transection in the Lesions of Multiple Sclerosis	10.1056/NEJM199801293380502	3,032
Lucchinetti et al. (28)	2000	Ann Neurol	Heterogeneity of multiple sclerosis lesions: Implications for the pathogenesis of demyelination	10.1002/1531-8249(200006)47:6 <707::AID-ANA3>3.0.CO;2-Q	2,157
Polman et al. (26)	2006	N Engl J Med	A Randomized, Placebo-Controlled Trial of Natalizumab for Relapsing Multiple Sclerosis	10.1056/NEJMoa044397	2,094
Jacobs et al. (34)	1996	Ann Neurol	Intramuscular interferon beta-1a for disease progression in relapsing multiple sclerosis. The Multiple Sclerosis Collaborative Research Group (MSCRG)	10.1002/ana.410390304	2,004
Duquette et al. (35)	1993	Neurology	Interferon beta-1b is effective in relapsing-remitting multiple sclerosis. I. Clinical results of a multicenter, randomized, double-blind, placebo-controlled trial. The IFNB Multiple Sclerosis Study Group	10.1212/wnl.43.4.655	1,996
Lennon et al. (39)	2004	Lancet	A serum autoantibody marker of neuromyelitis optica: distinction from multiple sclerosis	10.1016/S0140-6736(04)17551-X	1,940
Wingerchuk et al. (40)	2006	Neurology	Revised diagnostic criteria for neuromyelitis optica	10.1212/01.wnl.0000216139.44259.74	1,842
Liddelow et al. (29)	2017	Nature	Neurotoxic reactive astrocytes are induced by activated microglia	10.1038/nature21029	1,761
Maurano et al. (30)	2012	Science	Systematic localization of common disease-associated variation in regulatory DNA	10.1126/science.1222794	1,708
Sawcer et al. (31)	2011	Nature	Genetic risk and a primary role for cell-mediated immune mechanisms in multiple sclerosis	10.1038/nature10251	1,707
Ebers et al. (33)	1998	Lancet	Randomized double-blind placebo-controlled study of interferon β-1a in relapsing/remitting multiple sclerosis	10.1016/S0140-6736(98)03334-0	1,691
Kappos et al. (36)	2010	N Engl J Med	A placebo-controlled trial of oral fingolimod in relapsing multiple sclerosis.	10.1056/NEJMoa0909494	1,689
Chen et al. (32)	1994	Science	Regulatory T cell clones induced by oral tolerance: suppression of autoimmune encephalomyelitis	10.1126/science.7520605	1,621
Holick et al. (41)	2004	Am J Clin Nutr	Sunlight and vitamin D for bone health and prevention of autoimmune diseases, cancers, and cardiovascular disease	10.1093/ajcn/80.6.1678S	1,582

The article that received the most citations was the seminal work of Kurtzke et al. on rating neurologic impairment in patients with MS using an expanded disability status scale (EDSS) by 10,052 citations (20). John Francis Kurtzke (1926–2015) was a renowned neuro-epidemiologist and Professor of Neurology at Georgetown University, who authored more than 200 peer-reviewed articles. However, he is best known for developing the Disability Status Scale (DSS) in 1954 and EDSS in 1983. Since then, EDSS has become the most commonly used clinical scoring system to evaluate the overall functional disability of patients with MS, with a score ranging from 0 (normal) to 10 (death due to MS) in half-point increments. It has the merits of objectively displaying the differences in an MS clinical picture over time, which can be said for only a small number of scales (21).

The rest of the top five most cited articles discussed the diagnostic criteria of MS. The work of Poser et al., which proposed a new diagnostic criterion of MS in 1983, was second with 6,770 citations (22). Charles Poser and a group of American, Canadian, and British MS experts met in April 1982 for the purpose of developing new diagnostic criteria for MS, using the increasing availability of evoked potentials and neuroimaging in the late 1970's. The criteria were published in 1983 and consisted of two major groups, definite and probable, each with two subgroups: clinical and laboratory supported (22). The third article was the work of Polman et al. on revising McDonald diagnostic criteria of MS in 2011 with 5,428 citations (23), followed by the original famous diagnostic criteria proposed by McDonald et al. and published in 2001 in fourth place, with 4,985 citations (24). William Ian McDonald (1933–2006) was a professor of neurology at the Institute of Neurology of the University of London, England, who was the world's leading authority on MS in the second half of the twentieth century. The McDonald criteria were named after him as he directed an international panel in association with the National Multiple Sclerosis Society (NMSS). Two major changes were introduced: magnetic resonance imaging (MRI) criteria were incorporated into the scheme, and long-needed guidelines for the diagnosis of primary progressive MS were defined. The criteria underwent three revisions in 2005, 2010, and 2017, with the 2005 revision by Polman et al. coming in fifth place with 3,732 citations (25). It is worth mentioning that Chris Polman, a professor of neurology at VU University Medical Center, Amsterdam, Netherlands, and his colleagues published three articles of the top 10 articles; two on revising McDonald diagnostic criteria of MS in 2005 and 2011, and a randomized, controlled clinical trial of the use of natalizumab in MS (26). In a 2014 study, two papers published by Polman et al. received the most citations in MS research (7). A scale assessing fatigue severity in patients with MS by Krupp et al. came in sixth place with 3,218 citations.

Among the top 20 most cited articles, six articles evaluated the pathophysiological mechanisms in MS. The work of Trapp et al. (27) on axonal transection as the pathologic correlate of the irreversible neurologic impairment in MS came in 7th place with 3,032 citations. This was followed by the work of Lucchinetti et al. on the different patterns of demyelination in MS plaques (2,157) (28), Liddelow et al. on the neurotoxic role of reactive astrocytes in CNS (1,761) (29), Maurano et al. on the involvement of regulatory DNA variations in diseases (1,708) (30), Sawcer et al. on genetic risk in cell-mediated immune mechanisms in MS (1,707) (31), and Chen et al. on suppression of autoimmune encephalomyelitis by regulatory T cell clones, induced by oral tolerance (1,621) (32). These important studies had been a cornerstone in the current knowledge regarding fundamental pathophysiological processes behind the disease, which led to better disease diagnosis, classifications, and management. Another five articles investigated MS therapeutics, two articles on interferon β-1a (33, 34), one article for each of interferon β-1b (35), natalizumab (26), and fingolimod (36). These were randomized double-blind placebo-controlled trials that had been the result of a collaborative work of several international research groups, which eventually led to the U.S. Food and Drug Administration (FDA) approval of these therapeutics.

### Author Analysis

The list of the top 10 most influential authors in MS research is shown in Table 3, representing those with the highest numbers of total citations, in addition to h-index, g-index, and total number of publications. Interestingly, all of the top 10 came from European countries, with half of them coming from the UK and Italy. Six of them authored articles that were cited 30,000 times or more. Massimo Filippi holds the top position of the list in both the number of total citations (39,896), as well as the number of publications (522). Filippi M is a professor of neurology at Università Vita-Salute San Raffaele, Milan, Italy. His most cited work was titled “Diagnostic criteria for multiple sclerosis: 2010 revisions to the McDonald criteria” with 5,428 citations (23). In May 2010 in Dublin, Ireland, the International Panel on the Diagnosis of MS revised the 2005 version of the McDonald criteria, simplifying the criteria, and allowing for a more rapid diagnosis, with equivalent or improved specificity and/or sensitivity. The panel adopted new MRI criteria for dissemination in time (simultaneous asymptomatic gadolinium-enhancing and non-enhancing lesions on baseline MRI scans), and dissemination in space (i.e., at least one T2-lesion in two or more of the following CNS regions: periventricular, juxta-cortical, infratentorial, and spinal cord). Moreover, this revision improved the criteria's applicability to other populations (pediatric, Asian and Latin Americans).

**Table 3:** Top 10 authors with the highest number of total citations, also showing h-index, g-index, number of publications, and country.

**Author**	**Total citations**	**h-Index**	**g-Index**	**Number of publications**	**Country**
Filippi M	39,896	90	180	522	Italy
Miller DH	38,080	73	182	364	UK
Comi G	36,256	92	172	495	Italy
Kappos L	35,641	81	183	337	Switzerland
Thompson AJ	32,417	52	176	251	UK
Polman CH	30,563	57	171	249	UK
Hartung HP	28,908	89	168	231	Germany
Barkhof F	28,515	97	157	349	Netherlands
Montalban X	26,556	70	175	308	Spain
Olsson T	15,209	66	112	253	Sweden

The second author is Miller DH (38,080) with his paper titled “A randomized, placebo-controlled trial of natalizumab for relapsing multiple sclerosis,” receiving the highest number of citations with 2,094 citations (26). The data from this 2-year study (the AFFIRM study), in addition to data from (the SENTINEL study) demonstrated clear and dramatic reduction in MS clinical relapse activity, disability progression, and new MRI lesions, ultimately leading to the FDA approval of natalizumab for relapsing forms of MS. Comi G came third (36,256 citations), with a total of 1,707 citations for his most cited paper titled “Genetic risk and a primary role for cell-mediated immune mechanisms in multiple sclerosis” (31). This collaborative genome-wide association study (GWAS) involved 9,772 cases of a European descent, collected by 23 research groups from 15 different countries. The paper confirmed the major role of genes related to T-cell-mediated inflammation in the pathogenesis of MS through replicating almost all of the previously suggested susceptibility loci and identifying at least further 29 novel loci.

The following three authors in the list were Kappos L in the fourth place (35,641), followed by Thompson AJ in fifth (32,417), and Polman CH in the sixth place (30,563). Their article on revising the McDonald diagnostic criteria for MS in 2010, which was the same article by the first author, was their most cited work (23). Of the top 10 authors, four had <30,000 citations; Hartung HP came in the 7th place (28,908); Barkhof F in the 8th place (28,515); Montalban X in the 9th place (26,556); and Olsson T (15,209) in the 10th place. The work on genetic risk and the role of cell-mediated immune mechanisms in MS was the most cited for both Hartung HP and Olsson T, the same as Comi G (31). Furthermore, the 2010 revisions to the McDonald criteria were the most cited work for Montalban X (23).

The highest cited paper for Barkhof F was “Oral fingolimod or intramuscular interferon for relapsing multiple sclerosis” by 1,470 citations (37). This important phase III study was a 1-year, double-blind, double-dummy trial conducted on 1,292 patients by TRANSFORMS Study Group (ClinicalTrials.gov number, NCT00340834). It showed superior efficacy of oral fingolimod over intramuscular IFN-β1a as regards to relapse rates and MRI outcomes in patients with MS. This study, along with another 2-year, double-blind Phase III study (known as FREEDoMS), led to the FDA approval of fingolimod in 2010, as the first oral disease-modifying therapy. Finally, as regards to *h*-Index, Barkhof F was found to have the highest score (97), followed by Comi G (92), Filippi M (90), and Hartung HP (89). Despite its shortcomings, *h*-Index is an important tool in evaluating the output of an individual researcher, as well as providing an indication of the quality and consistency of the researcher's work by measuring the number of articles published and the number of citations received over time.

### Journal Analysis

The papers were published in 3,267 journals, of which 8 journals published more than 1,000 articles, as shown in Table 4. Unsurprisingly, the journal with the highest number of articles was *Multiple Sclerosis Journal* (*MSJ*) with 3,335 articles. *MSJ* (formerly *Multiple Sclerosis*) was first established in 1995, and as of 2020, it ranked 28 out of 208 journals in the category “clinical neurology,” The impact factor (IF) of *MSJ* has tripled since it was first included in the Journal Citation Reports (JCR), from 2.154 in 1999 to 6.312 in 2020. The journal is in the first quartile of their subject category (Q1), with an h-index of 131.

**Table 4:** The top journals in the field of MS research.

**Journal**	**Total articles**	**Total citations**	**h-index**	**Publication year start**	**Citation per article**	**Quartile**
*Multiple Sclerosis Journal*	3,335	103,608	131	1995	40.3	Q1
*Journal of Neuroimmunology*	2,003	63,074	140	1981	33.0	Q2
*Neurology*	1,531	99,242	364	1951	82.7	Q1
*Journal of the Neurological Sciences*	1,409	34,891	137	964	29.4	Q2
*Multiple Sclerosis and Related Disorders*	1,229	7,610	38	2012	6.2	Q2
Journal of Neurology	1,064	31,808	136	1974	29.89474	Q1
*PLoS ONE*	1,028	25,680	332	2007	24.98054	Q1
*Acta Neurologica Scandinavica*	1,016	23,625	95	1962	23.25295	Q1
*Journal of Neurology, Neurosurgery and Psychiatry*	787	42,581	206	1946	54.10546	Q1
*Journal of Immunology*	734	50,997	372	1976	72.7	Q1
*Annals of Neurology*	701	82,166	296	1977	139.1	Q1
*Brain*	607	73,186	336	1956	134.6	Q1

This was followed by *Journal of Neuroimmunology* (*n* = 2,003), a Q2 journal interested in publishing articles that involve immunologic methodology or fundamental immunology, with an IF of 3.478 in 2020. *Neurology*, the official journal of the American Academy of Neurology, came in third place with 1,531 citations. The journal is one of the most widely read and highly cited journals in the field of neurology, with an IF of 9.901 in 2020. *This was followed by two Q2 journals*: *Journal of the Neurological Sciences*, the official journal of the World Federation of Neurology, in the fourth place (*n* = 1,409), and *Multiple Sclerosis and Related Disorders* (*n* = 1,229), with an IF of 3.181 and 4.339, respectively.

Regarding the total number of citations, six journals exceeded 50,000 citations, with the greatest number for *Multiple Sclerosis Journal* (*n* = 103,608), followed by *Neurology* (*n* = 99,242). *Annals of Neurology* came in third place with 82,166 citations. This journal has a broad interest in the mechanisms and treatment of neurological diseases, with an IF of 10.422. It ranks 9th out of 208 journals in the category “clinical neurology.” This was followed by *Brain* (*n* = 73,186), *Journal of Neuroimmunology* (*n* = 63,074), and *Journal of Immunology* (*n* = 50,997). However, the journal with the highest number of citations per article among the top 50 articles was *The Lancet* (166.76), despite publishing only 148 articles with a total citation of 24,681. This high-impact general medical journal ranks second among 169 general and internal medicine journals globally (2020), with an IF of 79.321. This metric illustrates the importance of MS research and its impact on the scientific community. *Annals of Neurology* came in the second place (139.11), followed by *Brain* (134.573), and *Proceedings of the National Academy of Sciences of the United States of America* (97.09). *Brain* is a high-impact peer-reviewed neurology journal, founded in 1878 and published by Oxford University Press. It has an IF of 13.501 and ranks six out of 208 journals in the category “clinical neurology,” while *Proceedings of the National Academy of Sciences of the United States of America* is the official journal of the US National Academy of Sciences, which was published in 1914, with an IF of 11.205.

### Research Topics

MS literature has a broad spectrum of research fields, and most of the authors include their research topic in the document keywords. In this section, author keywords were analyzed to find the main trends in the different topics. The analysis of keywords can give valuable information about the themes of research. The evolution of keywords can inform us about the trends of past and future research, while analysis of the groups of keywords (clusters) can help us infer the main strands of MS research. Therefore, our analysis has included frequencies, trends, and keywords network with clusters.

### Keywords Frequency

Our dataset analysis shows that the most frequent keywords were multiple sclerosis (25,191), experimental autoimmune encephalomyelitis (EAE) (3,374), MRI (3,292), inflammatory (1,577), and demyelinating disease (1,507). The most common titles included patients with multiple sclerosis (2,253), EAE (2,158), relapsing remitting multiple sclerosis (1,178), central nervous system (784), progressive multiple sclerosis (701), and MRI (505). The rest of the top 20 keywords, titles, and abstracts are shown in Table 5.

**Table 5:** The top 20 most frequent keywords, keywords extracted from titles and abstracts.

**Modified keywords**	**Frequency**	**Title**	**Frequency**	**Abstract**	**Frequency**	**Original keywords**	**Frequency**
Multiple sclerosis	25,191	Multiple sclerosis patients	2,253	Multiple sclerosis MS	23,513	Multiple sclerosis	21,194
EAE	3,374	Experimental autoimmune encephalomyelitis	2,158	Central nervous system	6,846	Sclerosis	1,595
MRI	3,292	Relapsing_remitting multiple sclerosis	1,178	Experimental autoimmune encephalomyelitis	4,825	Multiple	1,517
Inflammatory	1,577	Central nervous system	784	Magnetic resonance imaging	3,956	MRI	1,242
Demyelinating disease	1,507	Progressive multiple sclerosis	701	Disability status scale	3,765	Demyelination	1,129
Autoimmune disease	1,465	Magnetic resonance imaging	505	Expanded disability status	3,751	Experimental autoimmune encephalomyelitis	1,018
Interferon	1,358	Experimental allergic encephalomyelitis	380	Autoimmune encephalomyelitis EAE	3,524	Magnetic resonance imaging	997
T-cells	1,349	Multiple sclerosis lesions	342	Nervous system CNS	2,690	Inflammation	925
Cognition	989	Myelin basic protein	274	Relapsing_remitting multiple sclerosis	2,278	Autoimmunity	875
CSF	933	Clinically isolated syndrome	261	Status scale EDSS	2,142	EAE	857
Cytokines	834	Secondary progressive multiple	226	Sclerosis MS patients	2,100	FATIGUE	815
Oligodendrocyte	832	Relapsing multiple sclerosis	206	Multiple sclerosis patients	2,010	Cerebrospinal fluid	750
Disability	825	Primary progressive multiple	205	Resonance imaging MRI	1,985	Disability	694
Fatigue	815	Remitting multiple sclerosis	197	Myelin basic protein	1,543	Depression	645
Interleukins	737	Randomized controlled trial	190	Cerebrospinal fluid CSF	1,540	Quality of life	613
NMOSD	715	Relapsing remitting multiple	186	Clinically isolated syndrome	1,158	Microglia	610
Depression	669	Neuromyelitis optica spectrum	178	Progressive multiple sclerosis	1,153	Cytokines	607
Optic nerve	645	Myelin oligodendrocyte glycoprotein	175	Multiple sclerosis RRMS	1,127	Cognition	605
Epidemiology	634	Pediatric multiple sclerosis	157	Myelin oligodendrocyte glycoprotein	1,085	Rehabilitation	604
Rehabilitation	629	Stem cell transplantation	147	Peripheral blood mononuclear	807	Epidemiology	594

### Keyword Co-occurrence Network Clusters

**Figure 3:**
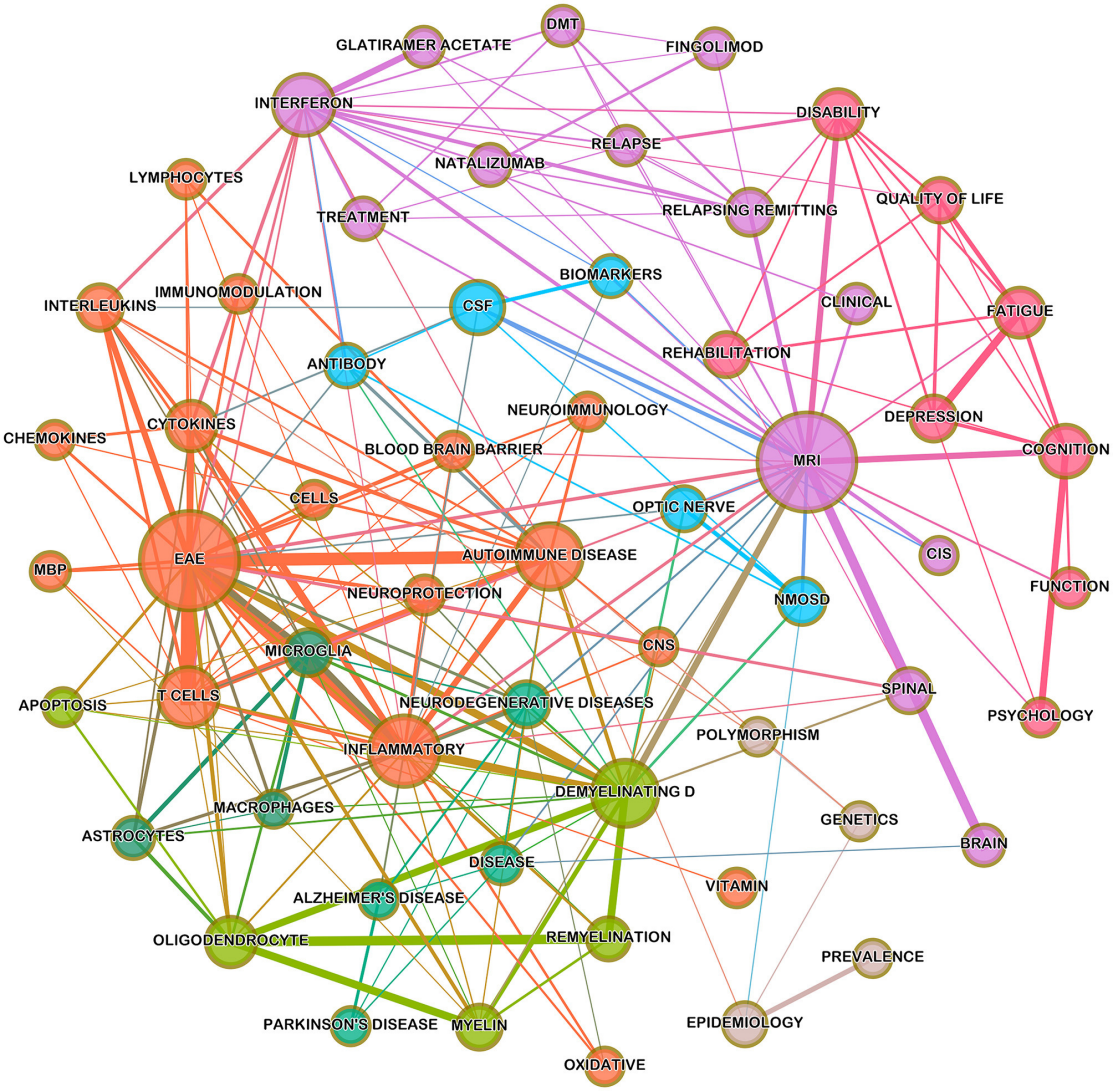
A co-occurrence network analysis map showing associations between keywords in the dataset, with the keyword “multiple sclerosis” removed. The size of the circle indicates the keyword count in the sample, line thickness indicates co-occurrence frequency, and colors indicate a cluster of keywords.

A keyword knowledge co-occurrence network was created, as visualized in Figure 3. The network shows a tightly knit group of clusters, representing the hotspots and main trends of research topics in MS over the previous eight decades based on the top authors' keywords. The larger the node is, the more significant the keyword will be, and the thicker the line is, the stronger the link would be.

The main research hotspots are summarized in the following themes.

#### Pathophysiology of MS

Further left on this map were the clusters representing the main pathophysiological mechanisms implicated in the development of MS (orange, light-green, dark-green) and included the following main keywords: “EAE,” “inflammatory,” “demyelinating disease,” “autoimmune disease,” “T-cells,” “lymphocytes,” “cytokines,” “chemokines,” “interleukins,” “blood-brain barrier,” “microglia,” “oligodendrocytes,” “astrocytes,” “macrophages,” “neurodegenerative disease,” and “oxidative.”

The largest node was the experimental autoimmune encephalomyelitis (EAE), which is the most commonly used experimental animal model for MS. It represents an interaction between a variety of immunopathological and neuropathological mechanisms that led to an approximation of the key pathological features of MS: inflammation, demyelination, axonal loss, and gliosis (42). This model is produced by administering a myelin basic protein peptide (MBP) fragment that induces an autoimmune response directed to the myelin sheath surrounding motor neurons (43). EAE was first described more than 85 years ago (44) and has been extensively used in MS research as a good model for understanding MS pathophysiological mechanisms. Furthermore, the use of EAE has expanded beyond the laboratory study of MS into the development of MS therapeutics as well.

The role of T-cells, cytokines, chemokines, and interleukins was evident in MS research, as illustrated by their node size and dense connections. T-cells play a key role in CNS inflammation through the regulation of a complex interplay between pro- and anti-inflammatory cytokines and interleukins, leading to the disruption of a blood-brain barrier and CNS demyelination (45). Several interleukins have been implicated in MS development, including IL-1β, IL-6, IL-17 TNFα (Tumor Necrosis Factor-α), and IFN-γ (Interferon-γ) (46). Although current evidence suggests that “B-cells” play a comparably important role to T-cells in CNS demyelination through differentiating plasma cells, producing autoreactive antibodies, and regulation of pro- and anti-inflammatory cytokines (47), this keyword was absent from the map. This could be related to the use of other keywords (e.g., lymphocytes, antibody) that could refer to B-cells, along with the relative recency of these emerging theories to be among the top keywords. Moreover, the role of activated macrophages and microglia in driving ongoing neurodegeneration was illustrated in the map, in addition to the role of oxidative injury as a major mechanism of both demyelination and neurodegeneration (48).

Interestingly, other neurodegenerative diseases, such as Alzheimer's disease (AD) and Parkinson's disease, were among the hotspots in the map. This could be explained through the pathophysiological mechanisms of neurodegeneration that affect these disorders. Chronic inflammation with microglia activation is also believed to play a major role in the formation of AD (49). In addition, studies have shown that MS could affect deep gray matter structures, leading to axonal loss in dopaminergic pathways, which could be responsible for the Parkinsonian features in such cases (50).

#### Neuropsychological Symptoms, Disability, Quality of Life, and Rehabilitation

**Figure 4:**
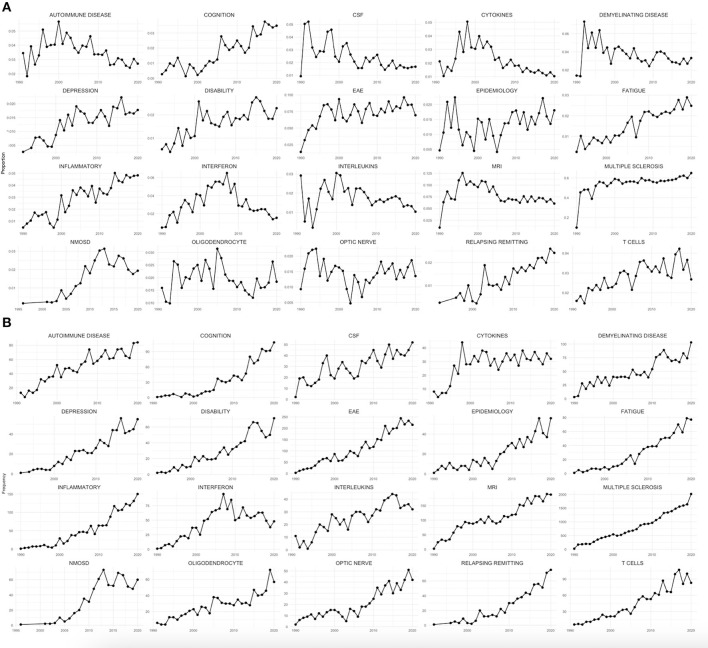
**(A)** The trend of the proportion of articles containing the top 20 most used keywords at each given year; **(B)** the trend of their frequency at each given year.

To the far right of the map, a cluster representing neuropsychological symptoms of MS is present (red), containing the following keywords: “depression,” “cognition,” “fatigue,” and “psychology.” They were also quite closely related to “disability,” “quality of life,” and “function.” This highlights the importance of non-motor symptoms and the increasing attention in MS research to their impact on disability and quality of life (QoL). Moreover, the trends in Figure 4 show that these topics are increasing in both frequency and trends, although their total fraction is still thin, ranging from the low of 0.5–2.5% of all MS articles.

The literature on cognition in MS has grown exponentially over the last 25 years, since Rao et al. (51) brought renewed attention to cognitive dysfunction as a core symptom of MS. Cognitive rehabilitation research is a nascent field, aiming at restoring cognitive function, or teaching compensatory strategies to attenuate the deleterious effect of refractory cognitive deficits on QoL (52). Fatigue, as one of the most common and troubling symptoms of MS, was found to be equally important as well. Evidence in literature showed a strong correlation between fatigue and physical functioning, disability, and QoL scores in patients with MS (53).

MS-related depression is another important clinical entity, with an estimated lifetime prevalence of more than 50%, and an annual prevalence of 20% (54). It took more than 100 years for researchers to turn their attention to the substantially negative impact of depression on QoL. The past two decades showed a growing trend of MS-related depression research; however, future studies still require a rigorous definition of a valid clinical phenotype of depression based on quantitative assessment, and constructing validity through brain imaging, immunological, and psychosocial research (55).

The keyword “rehabilitation” appeared among the top 20 in the analyzed studies; such findings are significantly different from the earlier bibliometrics which reported a negligible frequency of “rehabilitation” in their study (56). This could be partially attributed to the use of other keywords (e.g., functional assessment, disability) in earlier studies, besides the increasing attention to the value of rehabilitation in recent years.

#### Diagnosis of Multiple Sclerosis

The largest node of this cluster (purple) represents MRI, which can be seen densely connected to other clusters concerned with the pathophysiology and treatment of MS. It is also connected to other important diagnostic modalities, including “CSF,” “biomarkers,” and “antibody” (light blue). MRI was first introduced in the late 1970's, while the first MRI for a patient with MS was performed in 1981. Since then, this new technology has become an important paraclinical tool for diagnosing MS, monitoring therapeutic response, and in MS research (57). The main principle of MS diagnosis is based on showing dissemination of white matter lesions in space and time, and MRI was found to be the most sensitive method for revealing such dissemination, allowing for early diagnosis of CIS and MS and to rule out other differential diagnoses (58). Advanced MRI techniques, such as magnetization transfer imaging, spectroscopy, and functional MRI, have also been used in detecting tissue changes in MS, expanding our understanding of the pathogenesis of the disease (59).

In recent years, the use of the term “biomarkers” has shown a vast expansion in MS scientific literature, aiming at improving disease diagnosis, predicting disease progression, and improving clinical outcomes. Biomarkers currently in clinical use include oligoclonal bands (OCBs), MRI, JC viral titers, neurofilaments and GFAP in blood, and cerebrospinal fluid (CSF) (60).

#### Treatment of MS

At the top of the map (purple), there are four main disease-modifying therapies (DMTs): interferons, glatiramer acetate, natalizumab, and fingolimod. Although the pharmacological armamentarium for MS is currently expanding significantly, these drugs had been proved to be effective and well-researched throughout the past decades. In the 1980's, clinical trials were conducted to develop new MS treatments, and in the early 1990's, interferons and GA were approved as the first long-term treatments to affect the course of MS. IFNs impact the immune system in several ways, such as regulation of interleukins, and decreasing T helper (Th)-1 and Th17 production, which leads to an overall anti-inflammatory effect (61). Meanwhile, the mechanism of action of GA is not completely understood; however, most investigations have attributed its immunomodulatory effect to its capability to alter T-cell differentiation through promoting the development of Th2-polarized GA-reactive CD4 (+) T-cells (62).

In 2004, natalizumab was FDA approved as the first monoclonal antibody treatment for MS. It inhibits the migration of leukocytes into the brain, which results in reduced inflammation through blocking leukocyte α4 integrins binding to their endothelial receptors. In addition, fingolimod, a sphingosine-1 phosphate (S1P) receptor modulator, was the first oral DMT to be approved for MS. Both drugs had shown high-efficacy in clinical trials, compared to IFNs, with a relatively acceptable safety profile (63). However, more research is still needed to develop therapies for halting neurodegeneration, promoting remyelination, and promoting neuronal repair.

### Keyword Trends

Keyword trends explain the increase and decline of popularity in particular research areas. Figure 4 illustrates the absolute frequency (number) of the top 20 most used keywords and their relative frequency (share) throughout the years. Certain research topics have shown increase in frequency and in proportional trends (i.e., the ratio of the yearly articles), such as cognition, depression, disability, and fatigue. The growth of these research themes in recent years has indicated the increased awareness of MS researchers to the importance of the non-motor symptoms of MS and their impact on QoL. However, their increase constituted only 2.5% of the percentage of research, and more studies are still needed to optimize the overall outcome of patients with MS. Other research topics have declined over time such as interferons. The focus of research seems to have shifted away from interferons by the end of 2000's, coinciding with the development and approval of newer drugs with higher efficacy, although they are still used as active comparators in clinical trials (64). It is worth mentioning that a down trending keyword share is not synonymous with decreasing research, as the keyword may have become so prevalent that it is no longer necessary to use it as a keyword (e.g., autoimmune disease, demyelinating disease, and MRI).

## Discussion

Our bibliometric study on MS research spanned around eight decades, from 1945 to 2020, with a total of 48,356 articles. We offer a comprehensive and quantitative overview on the distribution of MS research by country, author, and journal during the assigned period of time. Moreover, we highlight the main themes and trends of research and how they evolved over time through reviewing the important keywords, keyword clusters, and seminal articles.

The number of published articles on MS has increased by 79.3% over the past 20-year period, with more than half of the scientific production published in the last decade. Although such a rate of growth is below the rate of all medical disciplines, which was around 96%, it is still higher than other specialties such as cardiovascular medicine, which had a growth rate of 64% during the same period (65). This notable growth may be a result of the increasing awareness of the burden of MS, the recent technological advances in diagnostic modalities, along with the significant increase in the number of registered clinical trials in recent years (66). Furthermore, we noticed increasing trends of research addressing the QoL of patients with MS, such as cognitive dysfunction, fatigue, and depression. Such themes of research have been barely visible within the earlier decades' studies, despite their huge impact on the disease outcome. Interestingly, only 5.3% of the documents in our dataset were not cited. This was found to be much lower than the reported percentage in 2012, which was 14.88% (7), reflecting the high impact and relevance of MS research in recent years.

Analysis by country affiliation of the primary author showed that the USA and European countries are still leading the research and the scientific collaborations on MS. The order of the top five countries remained the same when compared to what Aleixandre-Benavent et al. (7) reported in their bibliometric analysis in 2014, with the USA on top, followed by Italy, the UK, Germany, and Canada. The USA has produced a number of publications that are almost equal to the number of articles produced by the other four countries combined, with the majority being single-country publications. The number of articles published by Asian countries was found to be growing rapidly. China, for example, ranked 9th in MS scientific production, compared to the 25th place in 2012 (7). Besides population size, the socioeconomic status as illustrated by the gross domestic product (GDP) is one of the main factors related to research productivity. Countries with rapidly growing economies are expected to have more investment in research and subsequently contribute more to the growth of MS research (67, 68).

The number of articles published by countries from the MENA region has increased in recent years, which can be explained by the increase in disease prevalence in various MENA countries, as well as the recent advances in MS registries, allowing for nation-wide studies (69). It should be noted that some authors who are not native English speakers tend to publish their research in regional journals of their own language. This may explain why some big countries such as Russia do not appear among the top 25 producers of MS research, with most of their scientific production published in Russian language journals. Thus, their actual contribution to MS literature might be underrepresented in our analyses, especially as non-English articles are infrequently cited in international literature (70). As is common to most scientific fields, the knowledge we have at hand about MS remains heavily skewed toward the Western and industrialized countries (71). Therefore, we cannot claim in any credible way that we know the full breadth of MS as a disease in terms of pathology, symptomatology, or neuropsychological burden. A collaborative research that brings different countries together may help bridge the chasm in human knowledge.

The top 10 journals identified in the present study differed slightly from those reported in the bibliometric study between 2003 and 2012 (7), as *MSARD* (5th) and *Acta Neurologica Scandinavica* (8th) joined the list, while *Annals of Neurology* (11th) and *European Journal of Neurology* (14th) left. They contributed to 29.2% of the total number of publications. Although nine out of those 10 were neurological journals, MS research had been published in non-neurological subject areas as well, including but not limited to, general and internal medicine, psychiatry, biochemistry, and biology journals. MS requires a coordinated multidisciplinary collaboration of different medical specialties, which can be seen reflected on scientific publications. *Multiple Sclerosis Journal* still has the largest share of published articles with a contribution of 6.9% of all MS research, in addition to receiving the most citations.

The most cited original article was the famous work of Kurtzke et al. on assessing physical disability in patients with MS using EDSS (20), which has been used unchanged for more than 30 years, signifying its importance in clinical practice and in MS literature. Interestingly, this article was not among the top 20 in the earlier 2012 analysis (7), which could imply a recent trend in research that focuses on disability in the past decade, as a part of improving QoL and the general wellbeing of patients with MS, in addition to its well-known advantages as a standardization measure in clinical trials (72). However, EDSS has some documented weaknesses, including its heavy dependence on mobility, limited inter-and intra-rater reliability, insensitivity to changes in performing activities of daily living, and the lack of cognitive function assessment (73). As a result, several supplemental scales have been proposed to ameliorate these limitations in the last decade (74, 75).

According to the results of the co-occurrence network analysis, four highly connected clusters were observed. The main cluster, judged by its node size and connections, was the pathophysiological mechanisms of MS. Researchers have been extensively using the EAE model for more than 85 years, not only for better understanding of MS, but also for development of new therapeutic options (76). These studies have led to the generally accepted hypothesis that MS is mediated by pathogenic CD4+ T-cells, which secrete several proinflammatory interleukins against myelin antigens, followed by a broader neurodegenerative process (77). The next highly connected cluster was concerned mostly with the neuropsychological dysfunction in patients with MS. Despite its previous identification by Charcot (78), it has been overlooked for a long time. Our data over the past two decades showed that neuropsychological symptoms have been increasingly investigated, of which cognition, depression, and fatigue were among the most important (79). Furthermore, rehabilitation appeared among the top keywords in the analyzed data, which had been a neglected area of MS research as well (56). This increased awareness among researchers demonstrates the huge impact of non-motor problems on health-related QoL in patients with MS.

The other two connected clusters were concerned with the diagnosis and treatment of MS. It was not surprising to observe a large cluster formed around the keyword “MRI.” It has revolutionized the diagnosis of MS since the beginning of the 1980's and has been a cornerstone in MS diagnostic criteria. It has been widely used as an invaluable tool in understanding and monitoring disease activity in clinical trials and clinical practice (57). The recent interest in biomarkers was also observable in MS research, although this has not yet been translated in clinical settings.

The last cluster focused on the most important therapeutics based on their weight in literature. Our data showed that four DMTs (interferons, glatiramer acetate, natalizumab, and fingolimod) were the most studied MS drugs. Interferons and glatiramer acetate were the first approved drugs for MS; however, they have shown decline in the research trend in recent years. The era of high-efficacy drugs started with the approval of natalizumab (2004) and fingolimod (2010), and they have been a part of clinical practice and research since then (63).

However, our study demonstrated that some research gaps still exist in MS literature. First, most research came from Western countries, and although increasing recently, contribution from developing countries is still insufficient. Efforts should be made to support MS research in such countries for better understanding of the disease. Second, MS in special populations (the pediatric-age group, women during pregnancy, and the postpartum period) and MS in racial and ethnic minorities, who have distinct disease characteristics but historically low participation in clinical trials of DMTs, are still underrepresented. Furthermore, several new therapeutic options had less weight in MS research, despite being widely used in clinical practice, probably due to their relative recency. We hope that MS researchers can benefit from this analysis to fill these gaps in future studies.

A recent study that analyzed the top 100 articles that were discussed over social media has found the most discussed topics are related to the new treatment modalities and their side effects, while articles discussing pathology and symptomatology accounted for a fraction of the results. The authors recognized the particularity of social media in bringing topics of public concern into attention compared to traditional scientific venues, i.e., journals and conferences, which highlight academic community interests and opinions (9).

There are some limitations to the current study that should be addressed. First, this bibliometric analysis retrieved publications solely from the WoS database to include journals with impact factors. There are several other sources that were not included, e.g., research in other languages and emerging journals that are not indexed. In making this choice, we prioritized quality of the database, consistency of the meta-data, and credibility of the sources. Including other databases with no rigorous selection processes, e.g., Google Scholar, would risk the integrity of our results. Including data from rigorous databases would mix the citation counts from different sources, resulting in imbalanced metrics (80). Therefore, our results should be viewed and interpreted as representative of data published in venues with impact factors, and not necessarily representative of the full breadth of MS research (11). We reiterate the concerns and guidelines of interpreting bibliometrics data issued by the Leiden Manifesto, and therefore, we recommend our readers to interpret the results in the view of these guidelines (81). Bibliometric studies had a potential length time-effect bias, where the older articles receive more citations, and the long-standing authors are more likely to have published more; therefore, caution should be exercised when interpreting the results. Lastly, a bibliometric study is only as good as the meta-data; these are far from perfect and have known documented short-comings; while they do not affect the general conclusions, they are worth pointing out. Nonetheless, we have taken extra measures to clean the data to avoid these problems by cleaning the authors' names, keywords, etc.

In conclusion, the output of MS research has increased dramatically over the past 20 years, and analyzing these changes can provide important insights into the contribution of each country as well as international collaborations all over the world. Leaders of production of MS research were from the US and Western Europe; however, the number of articles published by Asian and MENA countries is on the rise. *Multiple Sclerosis Journal* had the highest number of articles and citations in MS research. The most cited article was by Kurtzke et al. on rating neurologic impairment in patients with MS using EDSS. Four main themes of research could be identified, focusing on understanding the pathophysiology of the disease, improving its diagnosis, and studying the efficacy and safety of the current and future treatments. Moreover, we see a rise of research themes addressing health-related QoL in recent years, with depression, cognition, and fatigue being the most studied. We hope that this bibliometric analysis can provide useful information for determining research and publication strategies in future investigations of MS.

## Author Contributions

III contributed to the concept, design, data collection, and draft of the manuscript. MS contributed to the concept, design, data analysis, interpretation, revision of the manuscript, and provided supervision. All authors provided critical feedback on drafts and approved the final manuscript.

## Conflict of Interest

The authors declare that the research was conducted in the absence of any commercial or financial relationships that could be construed as a potential conflict of interest.

## Publisher's Note

All claims expressed in this article are solely those of the authors and do not necessarily represent those of their affiliated organizations, or those of the publisher, the editors and the reviewers. Any product that may be evaluated in this article, or claim that may be made by its manufacturer, is not guaranteed or endorsed by the publisher.
